# Repetition suppression between monetary loss and social pain

**DOI:** 10.1186/s40359-024-01852-0

**Published:** 2024-06-18

**Authors:** Yue Zhang, Huixin Tan, Siyang Luo

**Affiliations:** Department of Psychology, Guangdong Provincial Key Laboratory of Social Cognitive Neuroscience and Mental Health, Guangdong Provincial Key Laboratory of Brain Function and Disease, Sun Yat-Sen University, Guangzhou, 510006 China

**Keywords:** Monetary loss, Social Pain, Repetitive suppression, FRN

## Abstract

The relationship between monetary loss and pain has been a recent research focus. Prior studies found similarities in the network representation patterns of monetary loss and pain, particularly social pain. However, the neural level evidence was lacking. To address this, we conducted an ERP experiment to investigate whether there is a repetitive suppression effect of monetary loss on the neural activity of social pain, aiming to understand if they engage overlapping neuronal populations. The results revealed that FRN amplitudes showed repetitive suppression effects of monetary loss on the neural activity of social pain. Our study suggests that monetary loss and social pain share common neural bases, indicating that they might involve shared neural modules related to cognitive conflict and affective appraisal.

**Supplementary Information:**

The online version contains supplementary material available at 10.1186/s40359-024-01852-0.

## Introduction

Like a versatile tool for distributing wealth, money wields a profound influence, fostering societal stability and progress and acting as a potent “psychological drug” that steers human thoughts and behaviors [1]. Specifically, money influences individuals’ mental states and behaviors through two common strategies: monetary loss and gain [2]. For example, by returning cash coupons to earn a five-star rating, merchants induce customers to change their feedback and perception of products by giving them monetary compensation (that is, monetary gain) [3]. On the contrary, though less explored, monetary loss also significantly influences individuals’ mental states and behaviors [4]. For instance, individuals report more negative emotions after monetary loss relative to monetary gains [5].

Monetary loss, which refers to the loss of money, often evokes a sense of pain [6]. Previous studies have found that the expectation and action of paying money to purchase products can cause direct and immediate displeasure, coined as the “pain of paying” [7]. The existence of pain for paying reveals the inseparable relationship between monetary loss and pain. Mazar et al. [8] conducted neuroimaging (fMRI) and behavioral studies to investigate the relationship between monetary loss and pain. Their findings suggest that paying with money activates brain regions linked to the emotional aspects of pain rather than the sensory pathways associated with sensory pain. However, current research on the interplay between monetary loss or gain and social incentives has primarily focused on reward-related stimuli. This emphasis is evident in studies investigating the neural mechanisms underlying monetary rewards and social gains, as well as those exploring reward processing in unhealthy populations such as depression or anxiety. These endeavors aim to advance targeted therapies for mental disorders [9–13]. Conversely, there exists limited research on negative incentives, with the majority of attention directed toward understanding the relationship between sensory pain and social pain [14, 15]. This focus has resulted in an oversight of the potential correlation between monetary loss and social pain.

Existing research concerning social pain and monetary loss has aimed to investigate how individual traits such as depression and anxiety influence the cognitive processing of both reward and loss stimuli [11, 16, 17]. However, these studies have often failed to adequately differentiate between the valence differences of negative and positive incentives, thereby overlooking the specific relationship between social pain and monetary loss. For instance, Nelson and Jarcho [11] found that correctly identifying stimuli resulting in monetary gain, monetary loss, social approval, and social disapproval feedback elicited Reward Positivity (RewP). Their results suggest that irrespective of the valence of the stimulus, the induction of RewP is contingent upon the accurate identification of such stimuli. Similarly, Sankar et al. [17] observed shared neural responses in the insula across both positive and negative stimuli, implying activations linked to the detection of salient information regardless of valence. Furthermore, He et al. [16] discovered that the valence of social stimuli influenced brain region activation in healthy individuals and those with subthreshold depression: individuals with subthreshold depression exhibited increased activity in the subgenual anterior cingulate cortex during anticipation of social loss. In contrast, activity in the putamen decreased during consumption of social gain compared to healthy controls. Unfortunately, they did not find differences in neural activation based on the valence of monetary stimuli, thus leaving the relationship between negative incentives (that is, monetary loss and social pain) unresolved.

Tan et al. [18] conducted a meta-analysis to explore the shared brain regions activated by monetary loss and pain, encompassing sensory and social pain. Their findings revealed that the neural network representation pattern associated with monetary loss exhibited more significant similarity to social pain than sensory pain, as indicated by the representational similarity analysis [18,19]. Sensory pain, as defined by the International Association for the Study of Pain (IASP), entails “an unpleasant sensory and emotional experience linked with, or resembling that linked with actual or potential tissue damage” [20]. In contrast, social pain is delineated as “the painful experience of actual or potential psychological distance from other people or social groups” from the core connotational perspective [15, 21]. On a broader extensional level, social pain is the unpleasant experience associated with actual or potential damage to one’s sense of social connection or social value (owing to social rejection, exclusion, negative social evaluation, or loss) [22]. To facilitate understanding and communication among scholars and enhance recognizability, the widely accepted terms “social pain” and “sensory pain” were employed in this study to represent all the subcategories that could cause social or sensory pain [13, 21–23].

Although Tan et al. [18] found shared neural bases between monetary loss and pain, whether we could extend this conclusion to the neural level remained unclear. Due to the relatively low spatial resolution of fMRI, the activation of a voxel can result from the activation of different neuronal populations within the same voxel [24]. Therefore, more evidence should be carried out to support the idea that shared neural mechanisms exist between monetary loss and pain.

Previous event-related potential (ERP) studies have found that monetary loss, relative to monetary gains, can elicit more negative feedback-related negativity (FRN) [25–27]. Moreover, a meta-analysis used the great grand averages method to confirm this main effect, suggesting that FRN might be a useful neural marker of monetary loss [28]. In addition, FRN is also a crucial neural marker of social exclusion. Kujawa et al. [29] indicated that rejection feedback (a signal of social exclusion) from peers was associated with negativity in the ERP wave compared to acceptance. Sun and Yu [30] further identified the role of FRN as a marker of social pain caused by rejection.

Therefore, this study aimed to investigate whether monetary loss and pain processing engaged overlapping neuronal populations to provide more persuasive evidence to support the neural correlates of monetary loss and pain by conducting an EEG experiment with a repetitive suppression (RS) paradigm. Considering that the neural representation of monetary loss was more similar to social pain than sensory pain [18], monetary loss and social pain would be focused. Specifically, we utilized the term “social pain” to denote the response caused by negative social evaluation in this study. RS effect refers to the decreased neural activity in response to a stimulus when this stimulus repeatedly occurs [31, 32], which has been reported at multiple spatial scales from neurons [33] to brain regions [34, 35]. When two successive stimuli elicit the RS of neural responses, these two stimuli are thought to activate overlapping populations of neurons [36]. Thus, testing the RS effect of monetary loss on the neural activity of social pain was a suitable method to examine whether the processing of monetary loss and social pain engaged overlapping neuronal populations. Drawing from prior research indicating a strong correlation between the FRN and both monetary loss and social pain [25, 29, 30, 37], our prior hypothesis was that there existed the RS effect of monetary loss on the neural activity of social pain, indexed by FRN. Furthermore, previous studies have identified variations in participants’ sensitivity to social evaluation, suggesting that such heterogeneity significantly contributes to differences in Feedback-Related Negativity (FRN) amplitudes [29, 30, 38–41]. For example, Cao et al. [38] showed that individuals with social anxiety disorder who were more sensitive to negative social feedback exhibited a larger FRN to positive social feedback than to negative social feedback compared to the healthy control participants; Van Der Molen et al. [41] further proved that the amplitudes of FRN were larger for anticipated social acceptance than for social rejection feedback in people with high levels of sensitivity of negative evaluation, compared with controls. Consequently, we further hypothesize that (1) The neural sensitivity to social pain elicited by the social evaluation task varied across participants; (2) the RS effect of monetary loss on the neural activity of social pain, as indexed by FRN, might vary across individuals with different sensitivities to social evaluation.

## Method

### Participants

We recruited 44 undergraduate students from Sun Yat-sen University (*M*_age_ = 19.57, *SD* = 1.54, 23 female). All participants were right-handed with normal or corrected-to-normal vision and had no history of neurological disorders. Our research was approved by the local ethical committee, and each participant provided informed consent before the experiment. Each participant was paid 100 Yuan for attendance and received a bonus based on their performance in the probabilistic learning task (ranging from 0 to 5 Yuan).

### Stimuli and procedure

**Fig. 1:**
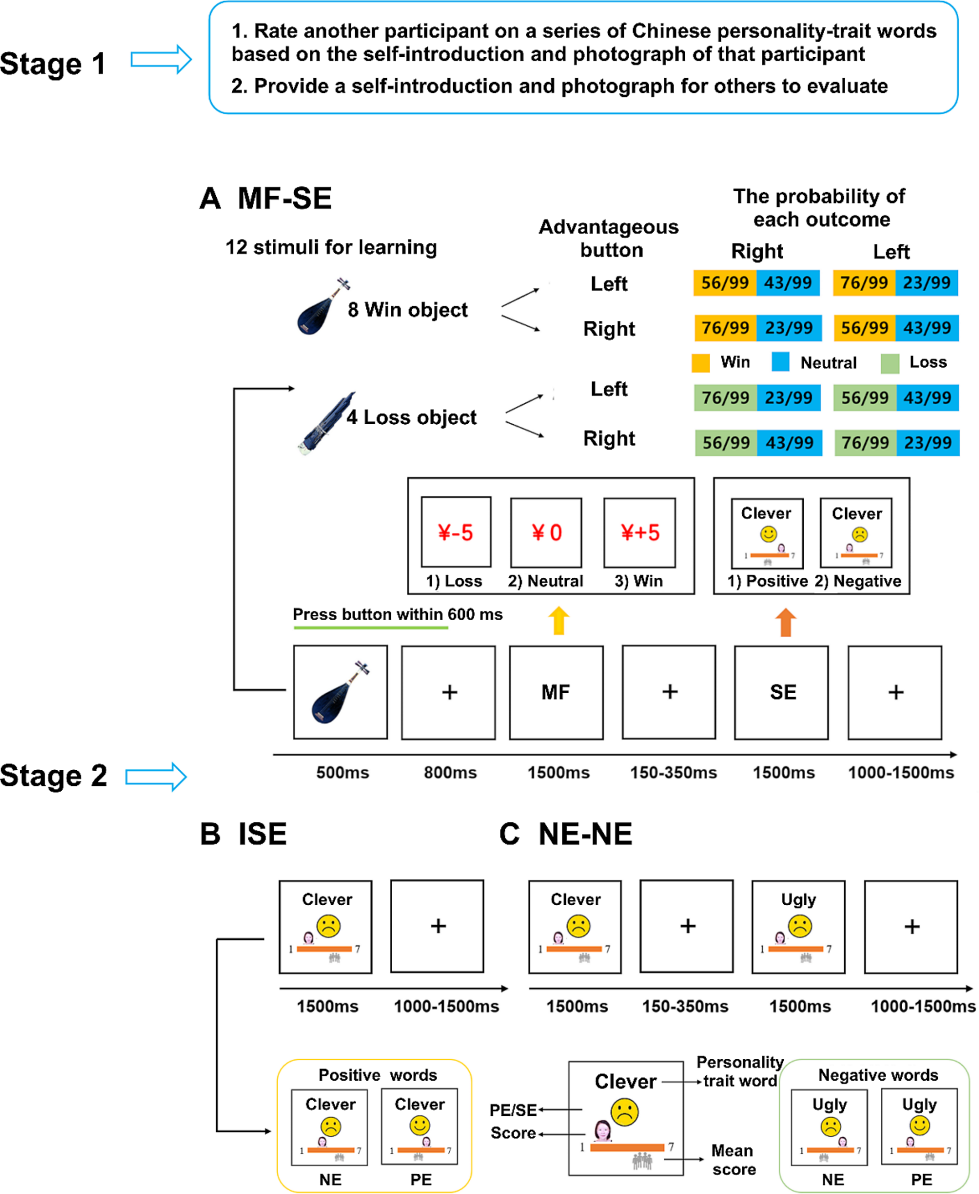
The Flowchart of the Experiment. **A**) The flowchart of the monetary feedback – social evaluation (MF-SE) trials. **B**) The flowchart of the independent social evaluation (ISE) trials. **C**) The flowchart of the negative evaluation – negative evaluation (NE-NE) trials. MF = monetary feedback; SE = social evaluation; PE = positive evaluation; NE = negative evaluation

The whole experiment consisted of two stages (see Fig. 1). Participants were told that this research was about cognitive processing in the first stage. Researchers selected the self-introduction and photograph of another participant before the experiment, and the current participant was required to rate that participant on a series of Chinese personality-trait words (“To what extent do you think clever is suitable to describe that participant?” 1 = absolutely not suitable, 7 = absolutely suitable). The whole sample of personality-trait words included 210 ordinary negative and positive personality-trait words with precise meanings; these words were selected based on previous study [42]. After that step, each participant completed the same self-introduction questionnaire [43] and was photographed by researchers for other participants to evaluate.

After completing Stage 1, participants were informed that they had finished the social evaluation task and provided personal information for evaluation by others. They returned for Stage 2 within 1–30 days (mean = 8). During Stage 2, participants received feedback based on others’ evaluations. Specifically, researchers briefly reminded participants of the experiment’s two-stage structure: “This experiment consists of two stages. In the first stage, you provided personal information for others to evaluate. We have collected evaluations from other participants, and after removing any invalid responses, the remaining ones will be presented to you during today’s ERP experiment.” During the ERP experiment, participants engaged in a probabilistic learning task [44]: one of twelve objects (e.g., a table) would be displayed on the screen, and participants needed to press the left or right button within 600 ms after the object was presented; this series of events was followed by monetary feedback (Win: ¥ + 5 / Neutral: ¥ 0 / Loss: ¥ -5). The images of all the objects were the same as in previous research [44]. Four of the twelve objects were randomly assigned to the win-object group, resulting in only neutral or monetary gain feedback; the remaining eight were assigned to the loss-object group, which resulted in only neutral or monetary loss feedback. For each group, half of the objects would be randomly mapped to the left button serving as the advantageous button, and the remaining half would be mapped to the right button serving as the advantageous button. For each group, the probabilities of two types of feedback corresponding to each button can be seen in Fig. 1. For each object, since participants were not informed of the probabilities of two kinds of feedback corresponding to each button, participants needed to use trial and error to maximize their total earnings of the experiment. To encourage fast button pressing, participants were told that a late response (later than 600ms) would lead to neutral feedback in the win-object trials and monetary loss feedback in the loss-object trials.

Between trials of the probabilistic learning task, participants received social evaluation feedback. Each feedback evaluation (see Fig. 1) would only correspond to one personality-trait word, and participants would be told of their scores and the mean of the whole population of participants. If the personality trait word was positive and participants’ scores were higher (lower) than the mean, the social evaluation would be positive (negative) with a smiling (distressing) face shown at the center of the screen. During the display of social evaluation feedback, participants only needed to view the screen without doing anything. Additionally, to encourage increased attention to social evaluation feedback, participants were told to take a test about their memory of the social evaluation feedback after the experiment. Unknown to participants, we presented negative and positive social evaluation feedback at a ratio of 7:3. We randomized the association between personality-trait words and types of feedback, and the proportion of negative or positive personality-trait words for each kind of feedback was controlled at a 50% level. We also randomized the order of two types of feedback with no more than five consecutive presentations of the same kind of feedback to avoid decreased sensitivity to social pain.

The experiment consisted of three types of trials (see Fig. 1): (1) a monetary feedback – social evaluation trial (360 trials; each object would be presented 30 times): an object would be randomly presented for 500 ms and participants needed to press the left or right button within 600 ms after object presentation; after a central fixation presented for 800 ms, adaptor monetary feedback would be randomly presented for 1500 ms, followed by the presentation of a fixation cross with a duration varying from 150 to 350 ms, and finally a target social evaluation would be randomly presented for 1500 ms; (2) an independent social evaluation trial (144 trials of positive evaluations and 112 trials of negative evaluations), individual social evaluation feedback was presented independently for 1500 ms; (3) an negative evaluation-negative evaluation trial (122 trials), an adaptor negative evaluation was randomly presented for 1500 ms and followed by the presentation of a fixation cross with a duration varying from 150 to 350 ms, after which a target negative evaluation was randomly presented for 1500 ms. Each trial ended with a fixation cross being presented with a duration varying from 1000 ms to 1500 ms. The entire experiment included ten runs, and the order of three types of trials was randomized with no more than three consecutive presentations of the same kind of trials to avoid the expectation effect. The display sizes of all social evaluation feedback and monetary feedback were kept constant across different conditions at a view distance of 70 cm. After completing this task, we randomly selected three trials and determined the payment bonus based on the highest monetary feedback received.

### EEG recording

We used the Neuroscan system to record EEG signals from 64 scalp electrodes arranged according to the International 10–20 system. We placed the reference electrode below the left mastoid and two additional electrodes on the left and right mastoids for offline reference. We recorded eyeblinks and vertical eye movements with electrodes placed above and below the left eye. The horizontal electrooculogram was monitored from the left and right external canthi electrodes. The impedance of all the electrodes was controlled below ten kΩ. We amplified EEG signals (bandpass 0.05–100 Hz) and digitized them at a sampling rate of 500 Hz for data acquisition.

### EEG preprocessing and analysis

We used MATLAB R2016a and EEGLAB 14.1.2b to preprocess the EEG data [45]. The EEG data were referenced offline to the average of the left and right mastoid electrodes and were filtered using a 1–30 Hz bandpass. We averaged the EEG data in each condition with epochs of 1000 ms (with a 150 ms prestimulus baseline). We interpolated electrodes and excluded epochs contaminated by motion (e.g., muscle movement), drift artifacts, and electromyographic artifacts to mitigate signal noise and artifacts. On average, 3 ± 2 electrodes were interpolated, and 4.49% ± 4.06% trials were discarded. Following artifact rejection, the number of trials for each condition was as follows: 136 ± 8 (independent positive evaluation, IPE); 107 ± 6 (independent negative evaluation, INE); 67 ± 13 (win); 159 ± 19 (loss); 114 ± 11 (monetary loss – negative evaluation, ML - NE); 109 ± 3 (negative evaluation – negative evaluation, NE - NE). After substituting abnormal electrodes and rejecting epochs with many artifacts, we used independent component analysis (ICA) to remove artifacts of eye movements or eye blinks [45, 46]. Specifically, any trial in which participants received monetary loss feedback, regardless of whether it resulted from a response timeout or pressing the disadvantageous button, and subsequently experienced social pain (negative evaluation), was categorized under the monetary loss – negative evaluation (ML - NE) condition. These trials were then subjected to further data analysis.

Based on previous studies, we averaged the mean amplitudes of FRN within a time window of 250-330ms after stimulus onset across the Fz, FCz, Cz, FC1, and FC2 electrodes [30].

Given that our experimental paradigm and analysis were designed to investigate the repetitive suppression (RS) effect of monetary loss on neural activity associated with social pain, we aimed to mitigate potential confounding factors caused by individual characteristics, such as the sensitivity to social evaluation, which could obscure such an RS effect. If participants exhibit insensitivity, any observed reduction in FRN amplitude expected from the RS effect might be mistakenly attributed to this insensitivity rather than the RS effect itself, leading to inaccurate conclusions. Therefore, to attenuate potential confounding effects, we calculated the participants’ sensitivities to social pain that operationalized by the disparity in FRN amplitudes between the IPE and INE conditions (ΔFRN_IPE−INE_) [38], and included this measure in further analyses.

To examine the neural response to monetary loss, a planned paired-t test was conducted to detect significant differences in FRN amplitudes between the Win and Loss conditions. We didn’t include the Neutral condition because the neutral feedback had double meanings: for win objects, the neutral feedback meant failing to win money; for loss objects, the neutral feedback meant succeeding in avoiding losing money. Since monetary loss can consistently elicit a more negative FRN [28], we predicted that monetary loss feedback would elicit a larger negative deflection of FRN for all participants.

We then focused on three different types of trials: (1) independent negative evaluation (INE), an individual negative evaluation occurred independently (no adaptor and only target NE); (2) negative evaluation – negative evaluation (NE-NE), two negative evaluations were presented in rapid succession (the preceding NE was the adaptor and the subsequent one was the target); and (3) monetary loss – negative evaluation (ML-NE), a negative evaluation was preceded by monetary loss feedback (the monetary loss feedback was the adaptor and the NE was the target). The RS effect of monetary loss on social pain was quantified by a smaller negative deflection of FRN in the ML-NE condition relative to that in the INE condition. If this RS effect was a partial RS effect, we would observe a smaller negative deflection of FRN in the NE-NE condition relative to that in the ML-NE condition; if this RS effect was an absolute RS effect, we would observe no significant difference in the amplitude of FRN between NE-NE and ML-NE condition.

To examine the RS effect of monetary loss on neural activity associated with social pain, we first conducted a two-way ANOVA that included trial types and participants’ sensitivities to social pain (ΔFRN) as two independent factors. Participants were categorized into two groups based on their ΔFRN values using a median split. Detailed methods and results can be found in the supplementary text. However, considering that the distribution of ΔFRN was not bimodal, transforming continuous variables into categorical variables using a median split can increase the risk of Type II errors due to the loss of statistical power (i.e., failing to detect an actual effect) and the risk of Type I errors by identifying false correlations [47]. This approach also results in the loss of detailed information about ΔFRN.

Therefore, we further conducted a linear mixed model analysis using R Studio version 4.3.1, employing the ‘lmerTest’ and ‘lme4’ packages. The dependent variable was the FRN amplitude, with fixed effects including sensitivity to social pain (ΔFRN), trial types of negative evaluation (INE/ML-NE/NE-NE), and their interaction. Subjects were included as random effects. For multiple contrasts analyzed within the same model, Bonferroni correction was applied to adjust for multiple testing. All reported statistical tests were two-tailed. The categorical variable (trial types of negative evaluation) was dummy coded, with ML-NE as the reference category. Continuous variables were normalized separately.

The linear mixed models were initialized with full model specifications based on recommendations from previous study [48]. In cases of non-convergence or overfitting, adjustments, such as model simplification, were implemented for random intercepts and slopes [48]. Initially, attempts were made to fit the full model, which included random effects of sensitivity to social pain (ΔFRN), trial types of negative evaluation (INE/ML-NE/NE-NE), and their interaction effects. However, the full model failed to converge. Subsequently, other models that included random effects of trial types of negative evaluation (INE/ML-NE/NE-NE) also failed to converge.

### The final model (model 0) that we focus was specified as follows

$$\begin{gathered} FRN\,amplitude \sim \,1 + \Delta FRN \\ *trial\,types\,of\,negative\,evaluation + (1\left| {subjects)} \right.\,\left( {{\mathbf{model}}\,{\mathbf{0}}} \right) \\ \end{gathered}$$

We then compared the performance of our final model (Model 0) with another model: Model 1. Model 1 included only the random intercept and random effect of sensitivity to social pain (ΔFRN), along with the fixed effects mentioned in Model 0. The results indicated that the Akaike Information Criterion (AIC) of Model 0 was smaller than that of Model 1 (Model 0: -171.2; Model 1: -167.7), suggesting that Model 0 provided a better fit. Therefore, the results of Model 0 were reported in the following Results section.

## Results

**Fig. 2:**
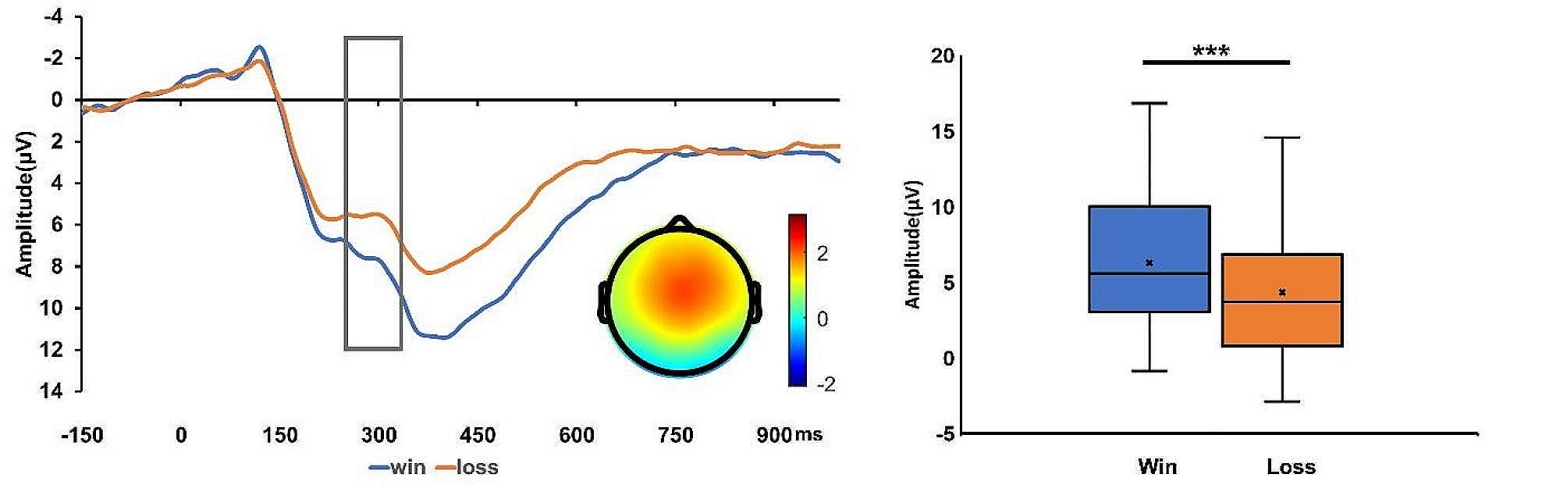
ERPs Recorded at FCz to Monetary gain or Loss conditions for all subjects. Note. The retangular area corresponded to the time window of FRN. The quartiles (boxes), means (X mark inside boxes), medians (horizontal lines inside boxes), and maximum and minimum excluding outliers (whiskers) are shown in the box plot. *p < 0.05, **p ≤ 0.01, ***p ≤ 0.001

The paired-*t* test between the Loss and Win condition (see Fig. 2, right panel) revealed that FRN was more negative in the Loss condition relative to the Win condition (*t* (43) = 4.99, *SE* = 0.37, *p* < 0.001, Cohen’s *d* = 0.81), indicating that monetary loss can consistently elicit a larger deflection of FRN.

**Fig. 3:**
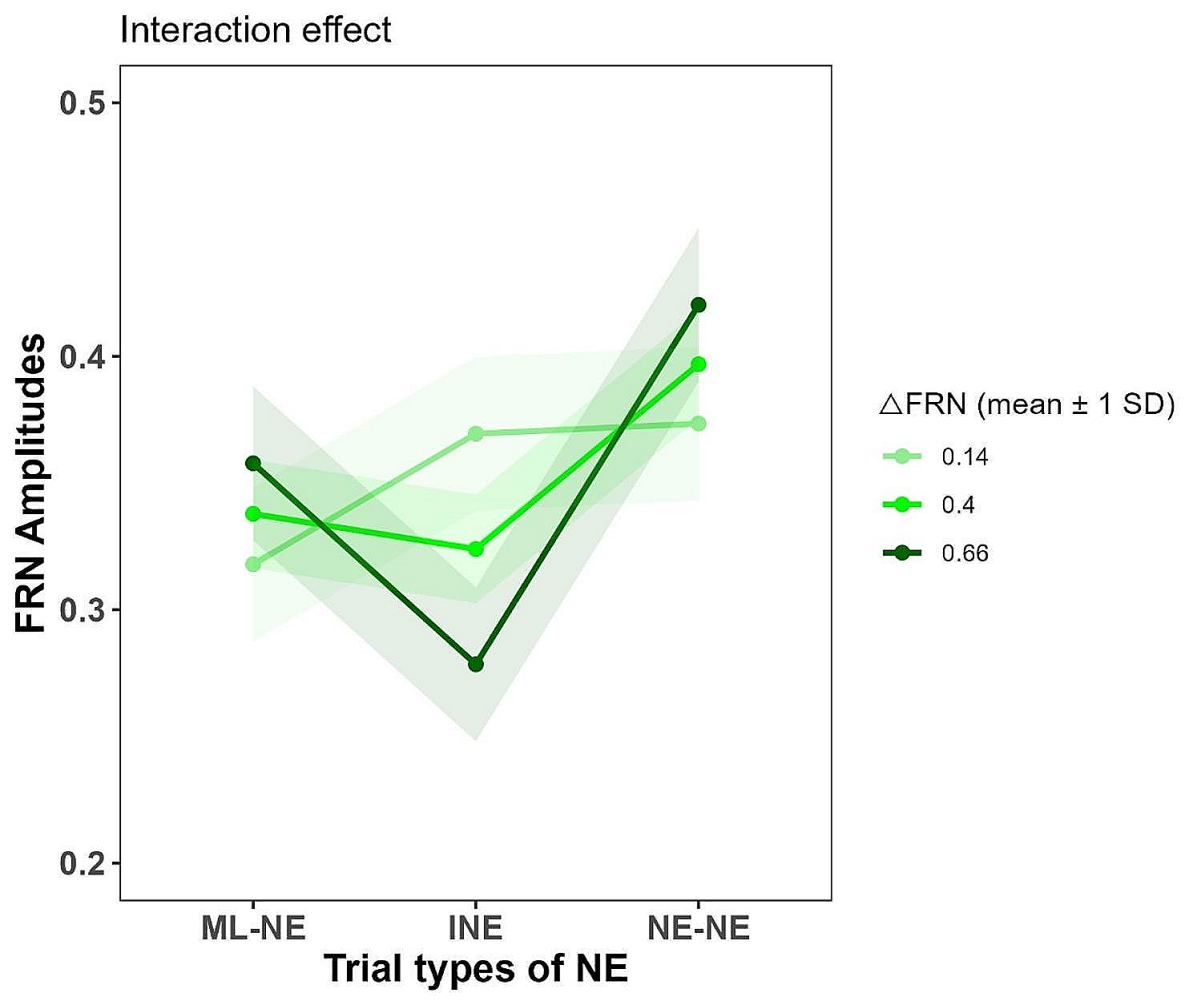
The interaction effect of linear mixed model. The light green, green, and dark green lines represent the low-sensitive, moderate-sensitive, and high-sensitive participants, respectively. The shaded areas indicate the 95% confidence intervals

We reported and interpreted the results of linear mixed model (see Fig. 3) based on the suggestion of Meteyard & Davies [49]. The effect of ΔFRN was statistically non-significant (${b}_{{\Delta }\text{F}\text{R}\text{N}}$= 0.08, 95% CI: [-0.09, 0.24], *t*(124) = 0.93, *p* = 0.354). The effect of trial type of NE-NE was not significant (${b}_{\text{N}\text{E}-\text{N}\text{E}}$ = 0.05, 95% CI [-0.02, 0.13], *t*(124) = 1.47, *p* = 0.145), while the effect of the trial type of INE was significant (${b}_{\text{I}\text{N}\text{E}}$ = 0.09, 95% CI [0.01, 0.16], *t*(124) = 2.37, *p* = 0.019). These results of trial types of NE suggested that target NEs elicited a similar negative deflection of FRN in the NE-NE condition relative to the baseline (ML-NE) condition while showing a significantly larger negative deflection of FRN in the INE condition relative to the baseline ML-NE condition. These results indicated that the processing of monetary loss and social pain engaged overlapping populations of neurons indexed by the RS effect of monetary loss on social pain, confirming the conclusion about the shared neural circuits between monetary loss and social pain.

**Fig. 4:**
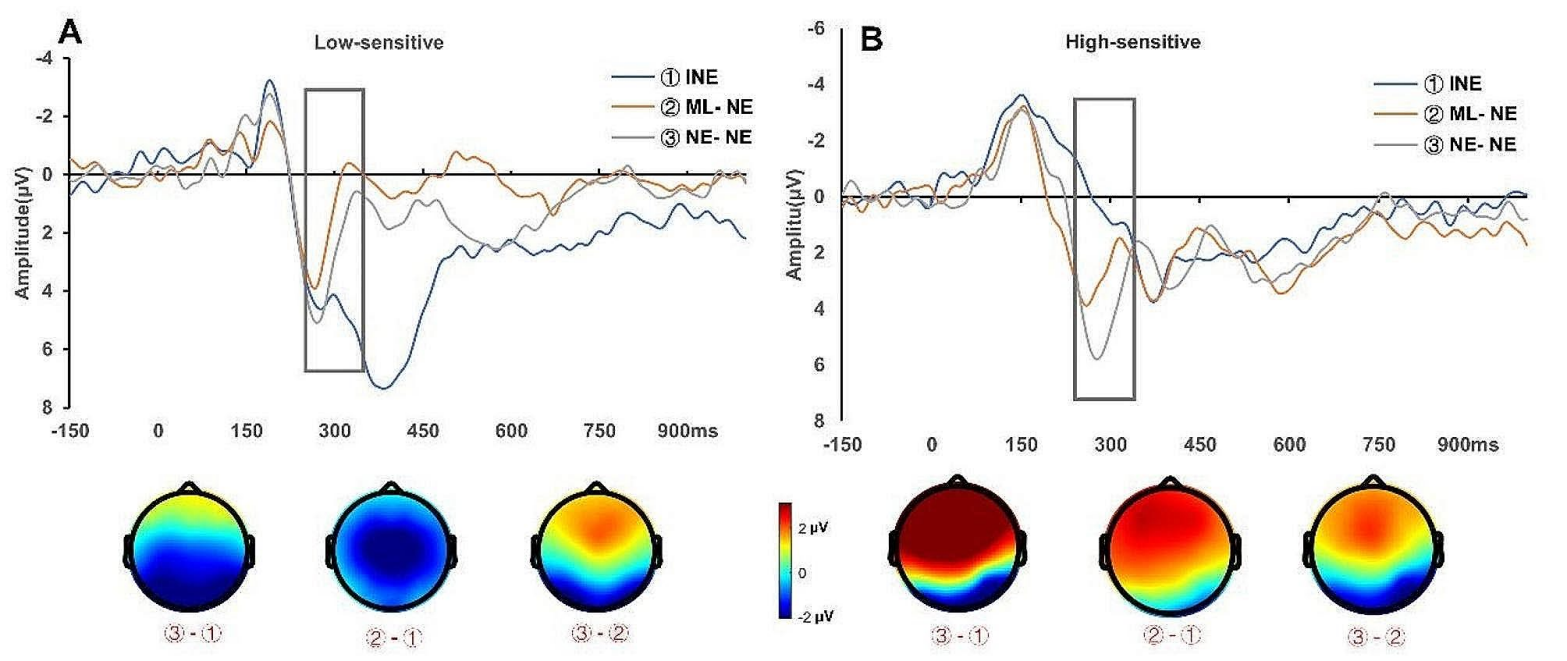
The Repetitive Suppression Effect of Monetary Loss on Neural Activity Associated with Social Pain. Panels A and B present ERPs recorded at FCz in response to target negative evaluations (NEs) for the low-sensitive group (**A**) and the high-sensitive group (**B**). These subgroups were selected to illustrate the interaction effect between participants’ sensitivity to social pain and the trial types, as identified in the linear mixed model analysis. The trial types include Independent Negative Evaluation (INE), Monetary Loss – Negative Evaluation (ML-NE), and Negative Evaluation – Negative Evaluation (NE-NE)

There was a significant interaction between ΔFRN and trial types of negative evaluation ($\text{I}\text{N}\text{E}:{b}_{\text{I}\text{N}\text{E}\text{*}{\Delta }\text{F}\text{R}\text{N}}$ = -0.25, 95% CI [-0.40, -0.10], *t*(124) = -3.27, *p* = 0.001; NE-NE: ${b}_{\text{N}\text{E}-\text{N}\text{E}\text{*}{\Delta }\text{F}\text{R}\text{N}}$ = 0.01, 95% CI [-0.14, 0.17], *t*(124) = 0.17, *p* = 0.862). Simple effect analysis demonstrated that ΔFRN modulated the impact of trial types of negative evaluation on the FRN amplitudes of target negative evaluations: For low-sensitive participants (mean - SD), trial types had no significant effect on the FRN amplitudes of target negative evaluations (INE: *t*(84) = 1.83, *p* = 0.07; NE-NE: *t*(84) = 1.96, *p* = 0.05), whereas for high-sensitive subjects (mean + SD), trial types significantly influenced the FRN amplitudes of target negative evaluations (INE: *t*(84) = -2.80, *p* = 0.006; NE-NE: *t*(84) = 2.21, *p* = 0.03)(see Fig. 3). For presentation purposes, we selected the high-sensitive participants (ΔFRN > mean + SD, seven subjects) and low-sensitive participants (ΔFRN < mean - SD, eight subjects) and plotted their average neural responses to these three types of trials to illustrate the pattern of our linear mixed model results (see Fig. 4).

These results demonstrated that the processing of monetary loss and social pain engaged overlapping populations of neurons indexed by the RS effect of monetary loss on social pain, confirming the conclusion about the shared neural circuits between monetary loss and social pain.

## Discussion

Money permeates every aspect of our existence, whether as a material medium for social exchange or a cultural representative symbol [1]. Pain is also an unpleasant feeling that everyone has experienced. Previous researchers have found that there are inseparable relationships between money and pain: the experience of social exclusion affected the willingness to purchase products [8]. For example, people who suffered from social pain sometimes spent more freely than others [50]; on the contrary, people’s willingness to accept pain enhanced when extra money was provided [51]. In contrast to the substantial attention directed toward investigating the effects of monetary rewards and social incentives [9–13], exploration of the relationship between monetary loss and pain has been comparatively limited. Existing studies have predominantly aimed to elucidate the influence of individual traits, such as depression and anxiety, on the cognitive processing of both rewarding and aversive stimuli [11, 16, 17], thereby inadequately clarifying the distinction between the valence differences of monetary loss and gain. Consequently, the relationship between social pain and monetary loss has remained unresolved. Tan et al. [18] conducted a meta-analysis and found that the network representation pattern of monetary loss was similar to that of pain, especially social pain. However, these results cannot be extended to whether monetary loss and pain processing engaged overlapping neuronal populations [18, 24]. Therefore, we conducted ERP recordings using the social feedback and probability learning task to examine this question. The results revealed that the processing of monetary loss and social pain engaged overlapping populations of neurons. Specifically, the repetitive suppression (RS) effect of monetary loss on the neural activity of social pain found in our study, which was indexed by FRN, suggests a shared neural circuit between monetary loss and social pain.

Consistent with previous studies, this study also found that monetary loss is related to FRN [6, 25, 37, 52]. We have identified a more negative FRN waveform under money-losing conditions than winning-money conditions. There are two possible explanations: 1) According to the reinforcement learning theory, FRN is related to the cognitive processing of stimuli’s feedback, reflecting that the current result is worse than expected [53]. Therefore, compared with winning money, the feedback of losing money may be a worse result than expected, so the FRN was more pronounced under money-losing conditions. In contrast, the alternative theory suggests that the FRN does not reflect the cognitive processing of feedback. Instead, it reflects the processes of assessing outcome events’ motivational/affective impact [2]. Hence, FRN would be more negative when losing money because losing money induced a negative emotional or motivational evaluation of outcomes.

However, this study found significant differences in participants’ sensitivity to social pain under the social feedback paradigm (see the paried t-test results between sensitive and insensitive subjects in the supplemetary text). These results are in the same vein as previous studies: Some studies have found that FRNs are sensitive to the valence of social evaluation: compared to positive evaluation, participants experienced significantly more negative FRNs when receiving negative evaluations [29, 30]. While other studies showed that FRN is not sensitive to social evaluation, there is no significant difference in the amplitude of FRN under positive or negative evaluations [38–40]. The observed heterogeneity in FRN responses may be attributable to variations in individual traits, as evidenced by previous research indicating that higher levels of social anxiety [38], depression [40], extraverted [54], anticipatory pleasure [55], behavioral inhibition predisposition [56] tend to elicit more pronounced FRN activity under conditions of social exclusion or unexpected reward or loss. However, our study did not incorporate additional assessments, such as surveys or ratings, capable of capturing such differences in individual characteristics, thus leaving the potential underlying causes of sensitivity differences unsupported. Future investigations should incorporate subjective or objective measures to elucidate the possible influence of such factors.

Furthermore, the results showed there exists the RS effect of monetary loss on neural activity in response to social pain. Based on the previous studies, the RS effect reflects that processing two similar stimuli that occur successively at very short intervals may involve the same neural circuits or neuronal population [34, 36]. Therefore, the RS effect indexed by FRN in this study reflected that monetary loss might share a common neural module related to cognitive processing or motivational or affective appraisal with social pain [36]. This interpretation supports previous studies that identified overlapping neural mechanisms between monetary and social incentives [11, 17]. More specifically, this RS effect might be related to the similarity in mental experience between monetary loss and social pain. While our study did not incorporate additional measurements to elucidate the potential causes of sensitivity differences, previous research has indicated that individuals with elevated levels of social anxiety [38] and depression [40] typically manifest increased concern and distress in response to social pain. Consequently, it is plausible that only individuals with heightened sensitivity (that is, high-sensitive subjects) experienced comparable mental responses between monetary loss and social pain, thereby contributing to the RS effect observed in our study. This result provided further evidence of the social resource theory of money, in which money was regarded as a social resource, similar to social relationships, which can elicit pain and a sense of security [57, 58]. Because of the similar role money plays like social relationships, the successive occurrence of money and social pain stimuli thus induced an RS effect in our experiment.

Additionally, we did not observe significant differences in FRN amplitudes between the NE-NE condition and the ML-NE condition. However, this result does not necessarily mean that monetary loss elicited absolute RS, instead of partial RS, of neural activity in response to social pain because the sample size was small; this small sample size may result in low statistical power to detect the difference in the FRN amplitudes between those two conditions [59]. Therefore, we remained unclear whether monetary loss elicited absolute RS or partial RS of neural activity in response to social pain. Future research needs to test the RS effect of monetary loss on neural activity of social pain in a larger sample.

## Conclusion

In conclusion, our research revealed that processing monetary loss and social pains shared the same neural circuits. Specifically, the RS effect indexed by FRN indicated the similarity in processing monetary loss and social pain at a more microscopic level by demonstrating that the processing of monetary loss and social pain engaged overlapping neuronal populations. Considering that most previous studies have primarily focused on monetary rewards and have explored similarities in how individuals process money rewards and social relationships, there has been a lack of sufficient exploration into the relationship between negative incentives, like the relationship between money loss and social pain. Therefore, our study indicated that not only in the field of monetary gain but also in the processing of money and social relationships in the field of loss shared similar neural bases.

### Electronic supplementary material

Below is the link to the electronic supplementary material.

Supplementary Material 1

## Data Availability

The datasets and materials during and/or analysed during the current study are available from the corresponding author on reasonable request.
