# Supplementary material for: Repetition suppression between monetary loss and social pain
Source: BMC Psychol. 2024 Jun 18;12:356. doi: 10.1186/s40359-024-01852-0 (PMC11186269; doi:10.1186/s40359-024-01852-0)
Supplement: Supplementary file 1 — Supplementary Material 1 [file 40359_2024_1852_MOESM1_ESM.docx]

Supplementary information

**Repetition suppression between monetary loss and social pain**

**Contents**

1. Distribution of ΔFRN……………………………………………………………...1
2. Methods and Results of the two-way ANOVA (with data split into two groups at zero)……………………………………………………………………………….1
3. Results of two-way ANOVA (split by the average value) ……………..…………8
4. **Distribution of ΔFRN**

We used the "ggplot2" package to draw the histogram to show the distribution of ΔFRN. Please see below:


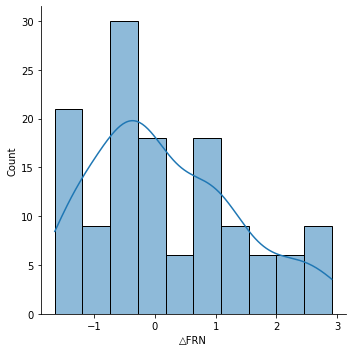


SFigure 1. Distribution of ΔFRN (IPE-INE).

We also calculated the skewness of ΔFRN and got a positive value (skewness: 0.52), which means it was a positive skew distribution.

1. **Methods and Results of the two-way ANOVA (with data split into two groups at zero)**

***Methods:***

Given that our experimental paradigm and analysis were designed to investigate the repetitive suppression (RS) effect of monetary loss on neural activity associated with social pain, we aimed to mitigate potential confounding factors caused by individual characteristics, such as the sensitivity to social evaluation, which could obscure such an RS effect. In cases where participants exhibited insensitivity, any observed reduction in FRN amplitude, expected as part of the RS effect, might erroneously be ascribed to this insensitivity rather than the RS effect itself, thus yielding inaccurate conclusions. Therefore, to attenuate potential confounding effects, we categorized participants based on their sensitivities to social pain, operationalized by the disparity in FRN amplitudes between the IPE and INE conditions (ΔFRN_IPE-INE_)(Cao et al., 2015). Specifically, we grouped subjects into two categories based on whether their ΔFRN values were positive or negative relative to zero, indicating their sensitivities to social evaluation. Since previous studies have shown that social pain can elicit more negative FRN (Kujawa et al., 2014; Sun & Yu, 2014), we assigned the participants with a positive ΔFRN_IPE-INE_ to the sensitive group (*N* = 22, *M*_age_ = 19.59, *SD* = 1.50, 11 females) and those with a negative ΔFRN_IPE-INE_ to the insensitive group (*N* = 22, *M*_age_ = 19.55, *SD* = 1.65, 12 females).

To verify the validity of grouping, we planned to conduct a paired-t test for each group to detect significant differences in FRN amplitudes between the Independent Positive Evaluation condition (IPE) and the Independent Negative Evaluation (INE) condition. We predicted that negative evaluations would elicit a larger negative deflection of FRN for the sensitive group. In contrast, for the insensitive group, negative evaluations would not elicit a more significant negative deflection of FRN.

To examine the neural response to monetary loss, another planned paired-t test was conducted for each group (sensitive/insensitive) to detect significant differences in FRN amplitudes between the Win and Loss conditions. We didn't include the Neutral condition because the neutral feedback had double meanings: for win objects, the neutral feedback meant failing to win money; for loss objects, the neutral feedback meant succeeding in avoiding losing money. Since monetary loss can consistently elicit a more negative FRN (Sambrook & Goslin, 2015), we predicted that monetary loss feedback would elicit a larger negative deflection of FRN for both groups.

We then focused on three different types of trials: 1) independent negative evaluation (INE), an individual negative evaluation occurred independently (no adaptor and only target NE); 2) negative evaluation – negative evaluation (NE-NE), two negative evaluations were presented in rapid succession (the preceding NE was the adaptor and the subsequent one was the target); and 3) monetary loss – negative evaluation (ML-NE), a negative evaluation was preceded by monetary loss feedback (the monetary loss feedback was the adaptor and the NE was the target). The RS effect of monetary loss on social pain was quantified by a smaller negative deflection of FRN in the ML-NE condition relative to that in the INE condition. If this RS effect was a partial RS effect, we would observe a smaller negative deflection of FRN in the NE-NE condition relative to that in the ML-NE condition; if this RS effect was an absolute RS effect, we would observe no significant difference in the amplitude of FRN between NE-NE and ML-NE condition.

Therefore, to examine the RS effect of monetary loss on the neural activity of social pain, a planned repeated-measures analysis of variance (ANOVA) was conducted on FRN amplitudes with the group (sensitive/insensitive) as a between-subjects variable and type of NE (INE/ML-NE/NE-NE) as a within-subjects variable. We predicted that the RS effect of monetary loss on the neural activity of social pain would occur in the sensitive group but not in the insensitive group due to the participants' differences in sensitivity to the social pain elicited by our task. We corrected the *p*-value using the Greenhouse-Geisser correction method when the data failed the spherical test. We used the Bonferroni post hoc test to conduct pairwise comparisons between types of NEs.

***Results***

The paired-*t* test of the sensitive group (see SFigure 2) revealed that FRN was more negative in the INE condition relative to the IPE condition (*t* (21) = - 6.10, *SE* = 0.19, *p* < .001, Cohen's *d* = - 1.30), indicating that negative evaluations elicited a larger negative deflection of FRN in the sensitive group. However, the paired-*t* test of the insensitive group (see Figure 2) revealed that no significant difference in the FRN amplitudes was detected between the INE condition and the IPE condition (*t* (21) = 7.79, *SE* = 0.10, *p* < .001, Cohen's *d* = 1.66), indicating that negative evaluations did not elicit a larger negative deflection of FRN in the insensitive group. Therefore, our approach of grouping participants was valid based on the above results.

The paired-*t* tests (see SFigure 3) revealed that regardless of the group, FRN was more negative in the Loss condition relative to the Win condition (Sensitive: *t* (21) = 4.12, *SE* = 0.50, *p* < .001, Cohen's *d* = 0.88；Insensitive：*t* (21) = 3.40, *SE* = 0.55, *p* = .003, Cohen's *d* = 0.72), indicating that monetary loss can consistently elicit a larger deflection of FRN.

ANOVA of the FRN amplitudes to target NEs (see SFigure 4) revealed a significant main effect of type of NE (*F* (1.73,72.69) = 7.44, *p* = .002，$\eta_{P}^{2}$ = 0.151) but not of the group (*F* (1,42) = 0.03, *p* = .870, $\eta_{P}^{2}$= 0.001). The interaction of group and type of NE was significant (*F* (1.73,72.69) = 7.16, *p* = .002, $\eta_{P}^{2}$= 0.146), as the simple effect of type of NE was significant in the sensitive group (*F* (2,84) = 11.56, *p* < .001, $\eta_{P}^{2}$0.216) but not in the insensitive group (*F* (2,84) = 3.03, *p* = .053, $\eta_{P}^{2}$= 0.067). The post hoc test results revealed that target NEs elicited a smaller negative deflection of FRN in the NE-NE condition relative to the INE condition (*t* (84) = 4.77, *SE* = 0.58, *p* < .001, Cohen's *d* = 1.34), suggesting that adaptor NEs elicited the RS of neural activity to target NEs. Also, target NEs elicited a smaller negative deflection of FRN in the ML-NE condition relative to the INE condition (*t* (84) = 2.90, *SE* = 0.58, *p* = .014, Cohen's *d* = 0.81), suggesting that adaptor monetary loss feedback elicited the RS of neural activity to target NEs. Moreover, no significant difference in FRN amplitudes was detected between the ML-NE and NE-NE conditions (*t* (84) = 1.88, *SE* = 0.58, *p* = .192, Cohen's *d* = 0.53).


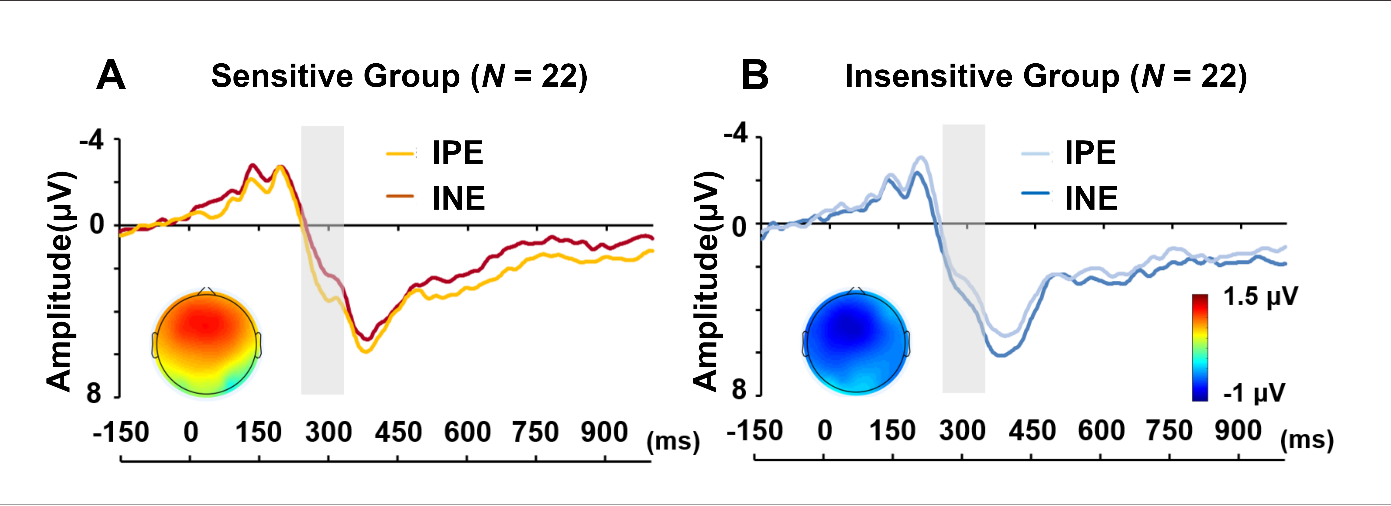


**SFigure 2.** ERPs Recorded at FCz to Independent Negative or Positive Evaluations

Note. A, B) ERPs recorded at FCz to independent negative or positive evaluations in the sensitive group (A) and insensitive group (B). IPE = Independent Positive Evaluation Condition; INE = Independent Negative Evaluation Condition. The shaded area corresponded to the time window of FRN.


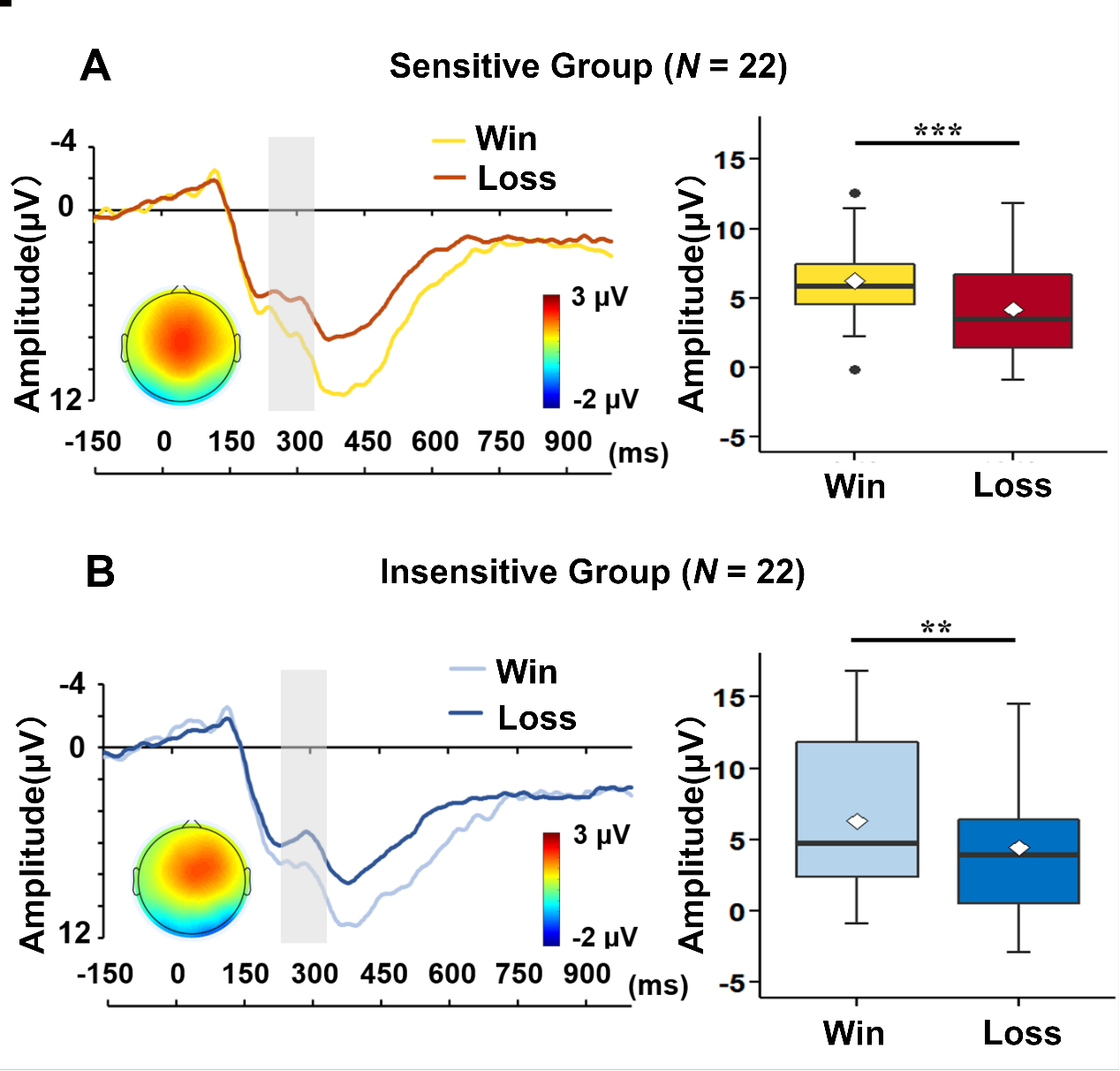


**SFigure 3.** ERPs Recorded at FCz to Monetary gain or Loss

Note. A, B) ERPs recorded at FCz to monetary gain or loss in the sensitive group (A) and insensitive group (B). The shaded area corresponded to the time window of FRN. The quartiles (boxes), means (diamond inside boxes), medians (horizontal lines inside boxes), maximum and minimum excluding outliers (whiskers), and outliers (data points higher or lower than 1.5-times the quartile, circles) are shown in the box plot. *p < .05, **p ≤ .01, ***p ≤ .001.

*
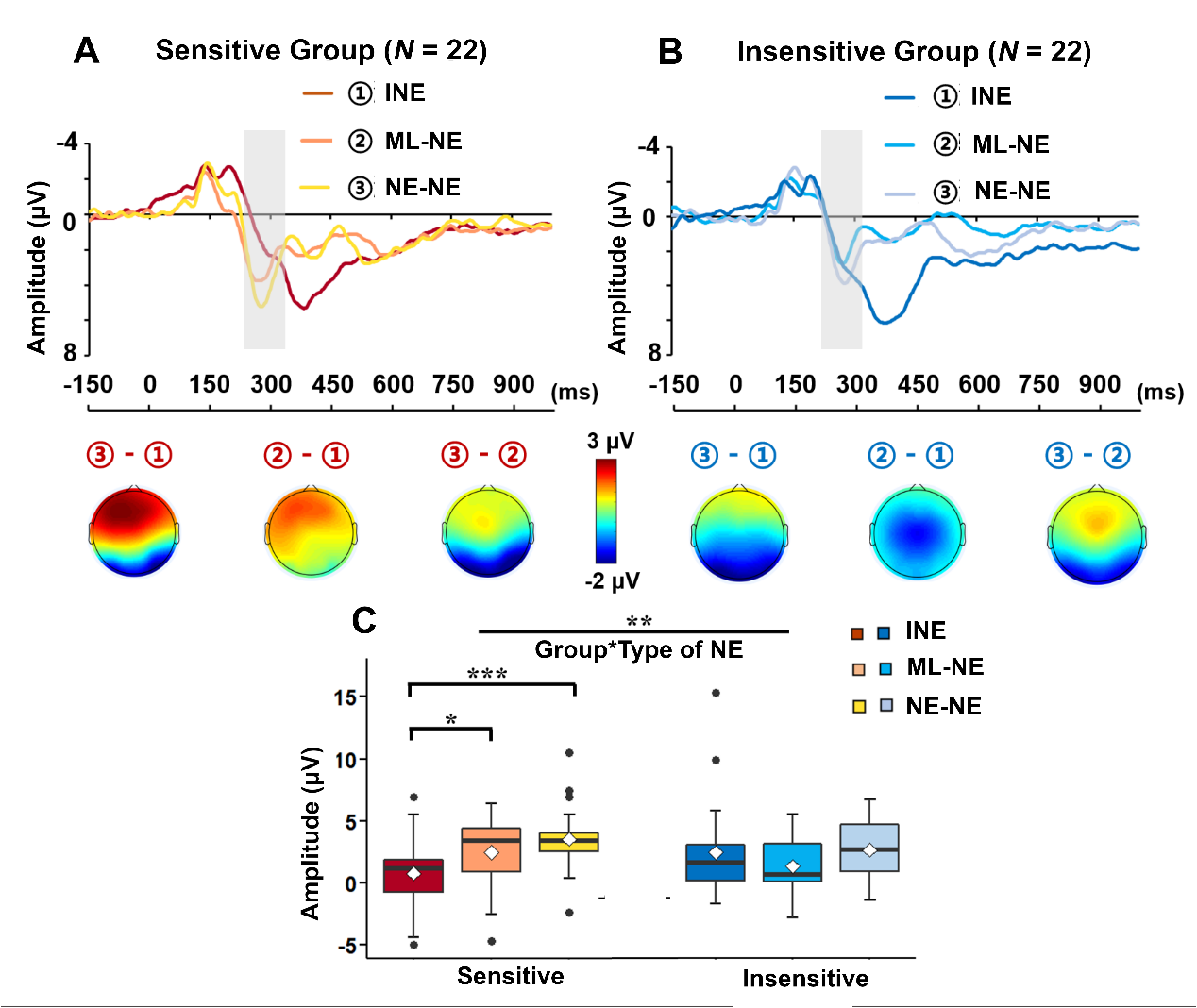
*

**SFigure 4.** The Repetitive Suppression Effect of Monetary Loss on Neural Activity of Social Pain. A, B) ERPs recorded at FCz to target negative evaluations (NEs) in the sensitive group (A) and insensitive group (B). C) The box chart about the FRN amplitudes of Independent Negative Evaluation (INE), Monetary Loss – Negative Evaluation (ML-NE) and Negative Evaluation – Negative Evaluation (NE-NE) condition. The quartiles (boxes), means (diamond inside boxes), medians (horizontal lines inside boxes), maximum and minimum excluding outliers (whiskers), and outliers (data points higher or lower than 1.5-times the quartile, circles) are shown in the box plot. *p < .05, **p ≤ .01, ***p ≤ .001.

1. **Results of two-way ANOVA (split by the average value)**

We also split our data by its mean value (0.17) of ΔFRN into two groups (high sensitive: 26 subjects / low sensitive: 18 subjects). We conducted the repeated-measures analysis of variance (ANOVA) on FRN amplitudes with the group (high sensitive/low sensitive) as a between-subjects variable and the type of NE (INE/ML-NE/NE-NE) as a within-subjects variable. The results showed that ANOVA of the FRN amplitudes to target NEs revealed a significant main effect of type of NE (*F* (1.73,72.67) = 8.43, *p* = .001，$\eta_{P}^{2}$ = 0.167) but not of the group (*F* (1,42) = 0.001, *p* = .970, $\eta_{P}^{2}$< 0.001). The interaction of group and type of NE was marginally significant (*F* (1.73,72.67) = 2.94, *p* = .066, $\eta_{P}^{2}$= 0.065), which showed a similar pattern to our previous ANOVA results, further indicating the robustness of our results in the main text.
